# Supplementary material for: Transocular sonography in acute arterial occlusions of the eye in elderly patients: Diagnostic value of the spot sign
Source: PLoS One. 2021 Feb 12;16(2):e0247072. doi: 10.1371/journal.pone.0247072 (PMC7880485; doi:10.1371/journal.pone.0247072)
Supplement: Attachment — Submitted filename: Response_To_Reviewers_letter_by_Hogrefe.pdf [file pone.0247072.s001.pdf]

Dear Dr. Czihal

Thank you for your request.

We hereby grant full permission to PLOS ONE to publish all content included in the manuscript „Transocular sonography in acute arterial occlusions of the eye in elderly patients: diagnostic value of the spot sign“ under the CC BY 4.0 license.

Previous publication of parts of this work (Vasa (2020), 49, Suppl. 105, p. 11, https://doi.org/10.1024/0301-1526/a000866) is not a peer-reviewed full publication; it rather represents a conference abstract referring to a preliminary oral presentation at a scientific meeting (4. DGA-Interventionskongress und 49. Jahrestagung Deutsche Gesellschaft für Angiologie – Gesellschaft für Gefäßmedizin e. V.).

Best regards,

Beatrix Marthaler
Production Editor

beatrix.marthaler@hogrefe.ch
+41 31 300 45 38

Hogrefe AG
Länggass-Strasse 76
3012 Bern
Schweiz
www.hogrefe.com/ch/
